# Evaluation of pericoronary adipose tissue attenuation on CT

**DOI:** 10.1259/bjr.20220885

**Published:** 2023-01-23

**Authors:** Runlei Ma, Roberto Fari, Pim van der Harst, Carlo N. De Cecco, Arthur E.Stillman, Rozemarijn Vliegenthart, Marly van Assen

**Affiliations:** 1 Department of Radiology, University of Groningen, University Medical Center Groningen, Groningen, the Netherlands; 2 Department of Radiology and Imaging Sciences, Emory University School of Medicine, Emory University, Atlanta, GA, USA; 3 Department of Cardiology, University of Groningen, University Medical Center Groningen, Groningen, the Netherlands; 4 University Medical Center Groningen, Data Science Center in Health (DASH), Groningen, the Netherlands

## Abstract

Pericoronary adipose tissue (PCAT) is the fat deposit surrounding coronary arteries. Although PCAT is part of the larger epicardial adipose tissue (EAT) depot, it has different pathophysiological features and roles in the atherosclerosis process. While EAT evaluation has been studied for years, PCAT evaluation is a relatively new concept. PCAT, especially the mean attenuation derived from CT images may be used to evaluate the inflammatory status of coronary arteries non-invasively. The most commonly used measure, PCAT_MA_, is the mean attenuation of adipose tissue of 3 mm thickness around the proximal right coronary artery with a length of 40 mm. PCAT_MA_ can be analyzed on a per-lesion, per-vessel or per-patient basis. Apart from PCAT_MA_, other measures for PCAT have been studied, such as thickness, and volume. Studies have shown associations between PCAT_MA_ and anatomical and functional severity of coronary artery disease. PCAT_MA_ is associated with plaque components and high-risk plaque features, and can discriminate patients with flow obstructing stenosis and myocardial infarction. Whether PCAT_MA_ has value on an individual patient basis remains to be determined. Furthermore, CT imaging settings, such as kV levels and clinical factors such as age and sex affect PCAT_MA_ measurements, which complicate implementation in clinical practice. For PCAT_MA_ to be widely implemented, a standardized methodology is needed. This review gives an overview of reported PCAT methodologies used in current literature and the potential use cases in clinical practice.

## Introduction

Inflammation plays a key role in the genesis of coronary artery disease (CAD), as suggested by the presence of immune cells in early phase atherosclerotic lesions.^1^ Studies have shown that approximately 60% of all myocardial infarctions occur in patients without significant coronary artery stenosis, caused by plaque or rupture of non-obstructive, presumably vulnerable, highly inflamed atherosclerotic plaques.^2,3^ Therefore, biomarkers that reflect this inflammation may be a potential early indicator of CAD risk. Pericoronary adipose tissue (PCAT), an indicator of coronary inflammation, has become a biomarker of interest, however, clinically available and validated measurements have been proven challenging. This review gives an overview of PCAT methodologies used in current literature and the potential use cases.

## Visceral adipose tissue

Adipose tissue is distributed throughout the human body; its main role is to store energy in the form of lipids, while it also shields and insulates tissue and organs. In the last decades, its critical role in endocrine signalling has been recognized. Adipose tissue can be categorized into white, brown, and beige fat, and can be classified into two main categories according to metabolic characteristics and location: subcutaneous adipose tissue (SAT) and visceral adipose tissue (VAT).^4^ VAT is the hormonally active component of total body fat, and produces molecules and hormones such as leptin, adiponectin, estrogen, resistin, and cytokines, which influence normal and pathological processes in the human body, both systematically and locally. In certain conditions, such as obesity, VAT mass can increase ectopic and may influence the susceptibility to comorbidities such as diabetes and atherosclerosis.

## The role of epicardial adipose tissue

Epicardial adipose tissue (EAT) is the VAT fat depot located between myocardium and visceral layer of the pericardium and covers 80% of the cardiac surface.^5^ Due to contact with myocardium, factors released from EAT have a direct paracrine effect on cardiomyocytes, and a potential role in the development ofCAD.^6^ These characteristics support the concept of EAT asa biomarker of CAD.^7–9^ EAT serves as a local regulator of free fatty acid (FFA) homeostasis, releasing FFAs into the coronary microcirculation and storing them depending on energy requirements. This adipose tissue is more sensitive to lipogenesis than other VAT compartments,^10^ and can release or uptake FFA at a higher rate than other fat depots while under metabolic stress.^11^ Pathological conditions, such as obesity and diabetes, but also genetic and environmental factors, may drive the shift towards dysfunctional EAT characterized by a pro-inflammatory and pro-atherosclerotic phenotype.^12^ In these conditions EAT becomes hypertrophic, leading to failure of triglyceride storage, increased lipolysis and inflammation.^5^ The role of vascular inflammation in the development of coronary atherosclerosis and rupture of vulnerable plaques, resulting in acute coronary syndrome (ACS), has long been postulated.^13^ One study supports the association of dysfunctional EAT with coronary inflammation and plaque severity^14^; while another study even suggests a strong relation between EAT and plaque vulnerability features.^15^ CT allows calculation of EAT volume, which is by now a known marker to estimate cardiac inflammation in relation to CAD severity and risk of adverse cardiac events.^16^

## From EAT to PCAT

**Figure 1.**
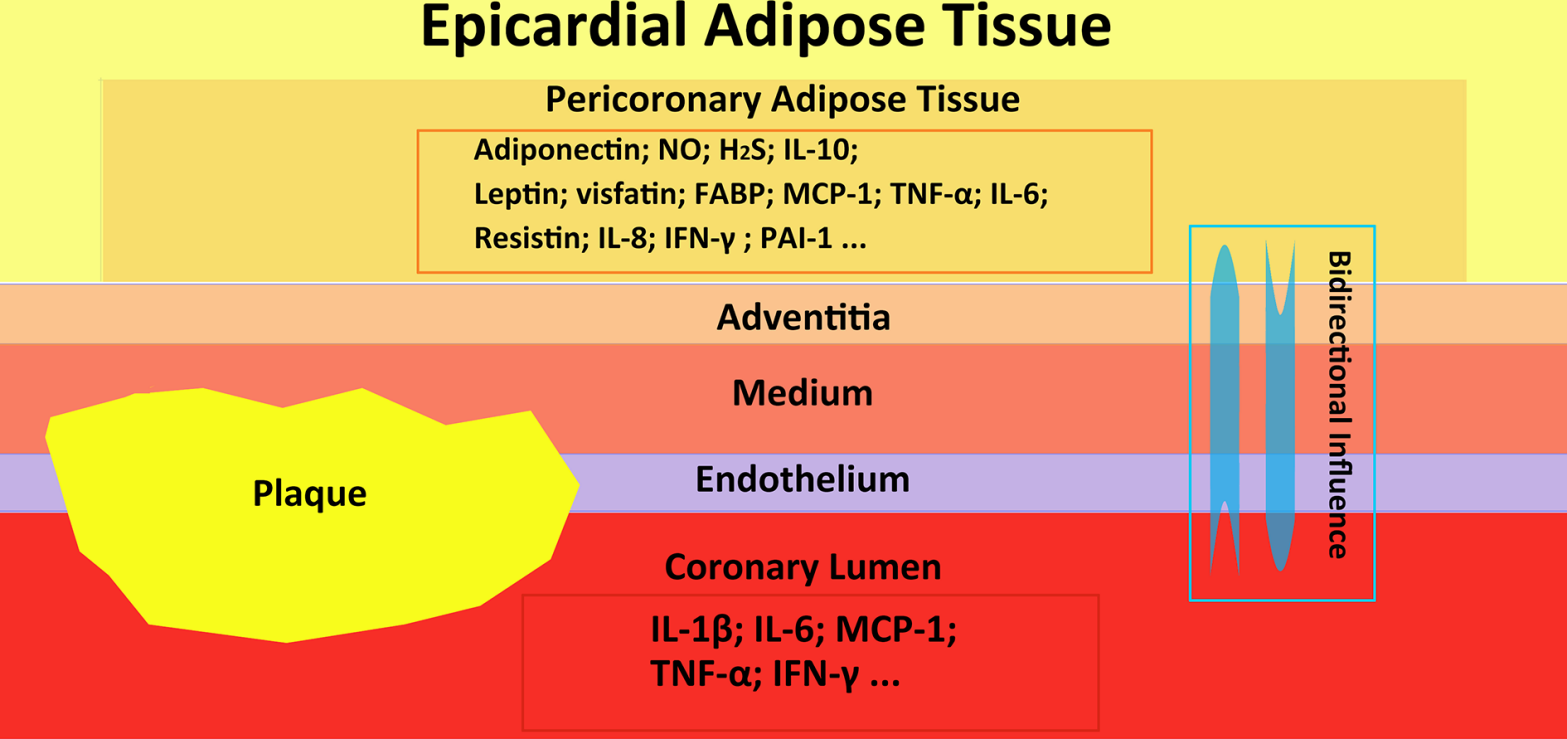
This figure shows the epicardial adipose tissue, pericoronary adipose tissue, and coronary artery with plaque. Besides that, the interaction of inflammatory factors among EAT, PCAT and coronary artery is shown. FABP, fatty acid binding protein; H2S, hydrogen sulfide; IFN, interferon; IL, interleukin; MCP, monocyte chemoattractant protein-1; NO, nitric oxide; PAI, plasminogen activator inhibitor; TNF, tumor necrosis factor.

Currently, there are no published recommendations for the standardization of EAT measurements.^17^ Some studies analyzed EAT linear thickness on transthoracic echocardiography, CT oMRI.^18,19^ EAT 3D volume quantification on cardiac CT is considered the most accurate measurement with the most literature describing its value.^17^ Nevertheless, EAT volume as measure of cardiac inflammation has some major limitations. Systemic influences, such as obesity and diabetes, as well as medications and even season can affect EAT and EAT volume.^20^ These systemic influences may cause differences between populations, or reflect temporary systemic variations, instead of coronary inflammation. The need of a more specific imaging marker, related to coronary inflammation, has recently turned attention to PCAT. PCAT is defined as the adipose tissue within the EAT depot that surrounds the coronary arteries,^21^ and has therefore the closest interaction with the adjacent coronary arteries,^22^ see Figure 1. Recent studies have shown that there is a bidirectional, biochemical communication between the coronary arterial wall and PCAT.^23^ When PCAT becomes dysfunctional it can produce biologically active factors that induce endothelial dysfunction and inflammation, leading to the progression of atherosclerosis.^24^ Vice versa, signals originating from the coronary wall can affect PCAT in a paracrine manner and lead to decreased perivascular lipid accumulation, with decreased lipophilic content and smaller, undifferentiated, lipid-poor adipocytes.^25^ These factors result in volume and attenuation difference of the PCAT depot, which can be evaluated using coronary CT angiography (CCTA).^26^

Although PCAT is included in the EAT depot, they have different pathophysiological effects and clinical significance. Compared to EAT, PCAT, in closer proximity to the coronary arteries, consists of a higher percentage of pre-adipocytes which are smaller in size with increased adipogenic gene expression compared to mature adipocytes. Inflammatory factors involved in the coronary atherosclerotic process prevent pre-adipocytes to mature into adipocytes in PCAT by paracrine effects.^26^ This suggests that PCAT is more influenced by the paracrine inflammatory signals from the coronary arteries than autocrine signals released from the fat depot itself, whereas EAT captures both paracrine and autocrine signals.^26^

## PCAT measurement methodologies

Several measurement methods for CT evaluation of PCAT have been reported in literature: thickness, volume and attenuation, the last one being the most commonly used.^27^ Most studies define PCAT as adipose tissuewith a 3 mm width around the coronary artery wall, usually the right coronary artery (RCA). There are different approaches to define the length and location of PCAT measurements. Below, we will describe the methodologies of PCAT from current literature.

## PCAT thickness

Previously, PCAT has been measured as thickness of the adipose tissue around the coronary artery. The most common way to measure PCAT thickness is by calculating the maximum EAT width around the proximal coronary artery in cross-sectional CT images. The limited number of CCTA studies on PCAT thickness on axial views or multiplanar reformats showed good reproducibility, and reported improved metabolic syndrome and atherosclerosis diagnosis in CAD patients.^24,26^ PCAT thickness measurement is a straightforward and fast approach. However, PCAT thickness contains less information than other PCAT variables, and sometimes it is difficult to visually identify and distinguish PCAT and EAT thickness.

## PCAT volume

PCAT volume is rarely used and subject to controversy since it is hard to define the measurement range of PCAT volume. There are several ways to calculate PCAT volume. One way is to calculate the volume by using the original method of PCAT_MA_ segmentation, so the volume of the 3 mm around the coronary arteries in CCTA.^28^ Another way is manually tracing the region containing PCAT in axial images perpendicular to the center line of the coronary artery, and subsequently summing every slice to create one volumetric measurement in CCTA.^29^ A third way is to manually define a region of interest according to coronary segments, and use the 3D reconstructions to calculate PCAT volume in non-contrast cardiac CT.^30^A recent study showed that RCA-based PCAT volume on contrast and non-contrast images is highly correlated.^28^ PCAT volume provides additional 3D information of PCAT compared to thickness. However, PCAT volume measurements have not been standardized and differences in measurement approached as described above could lead to different results.There is controversy about the association of PCAT volume with CAD, and outcomes of the few available studies show conflicting results.^26,30^

## PCAT mean attenuation

Nowadays, the most widely used PCAT measurement method is the mean attenuation of PCAT (PCAT_MA_). Here, the HU values of voxels in surrounding adipose tissue (defined as −190 to −30HU) perpendicular to the center line of the coronary artery in 3D reconstruction are averaged.^26^ Usually, the adipose tissue around the proximal coronary artery is measured with 40 mm length and 3 mm width, leaving a 1 mm gap around the coronary artery wall to avoid blooming effects of contrast medium.

PCAT_MA_ is an indirect measure of adipocyte size and lipid content, reflecting inflammation status. In contrast to PCAT volume, the role of PCAT_MA_in coronary inflammation has been proven in pathophysiological studies with histological samples.^26^, with relation to vulnerable lesions. Moreover, PCAT_MA_measurements have higher inter- and intrareader agreement, compared to PCAT thickness or volume.^28,31,32^ However, one study showed that variation in contrast intensity of CCTA images affects PCAT_MA_ assessment and that lumen-normalization might be a requirement for accurate PCAT_MA_measurements.^33^ Per patient, per vessel or per lesion PCAT_MA_ measurements have been proposed depending on the application of PCAT (Table 1 for overview).

**Table 1.** PCAT evaluation methods

PCAT measurements	Measured vessels	Start point	Length	Width	Gap between vessel wall
					
Patient-based	Original PCAT: RCA. Average of LAD, LCX and RCA	RCA:10 mm from ostium LAD and LCX: left bifurcation point	40 mm	3 mm 1 mm	1 mm
					
Vessel-based	RCA LAD LCX	RCA: 10 mm from ostium LAD and LCX: left bifurcation point LAD: 10 mm from bifurcation	40 mm 10 mm 5 mm	3 mm 1 mm	1 mm
					
Lesion-specific	RCA LAD LCX	Beginning point of the lesion centered around lesion mid-point	10 mm Lesion-length	3 mm 1 mm	1 mm

LAD, left anterior descending artery; LCX, left circumflex artery; PCAT, pericoronary adipose tissue; RCA, right coronary artery.

## Patient-based measurements

**Figure 2.**
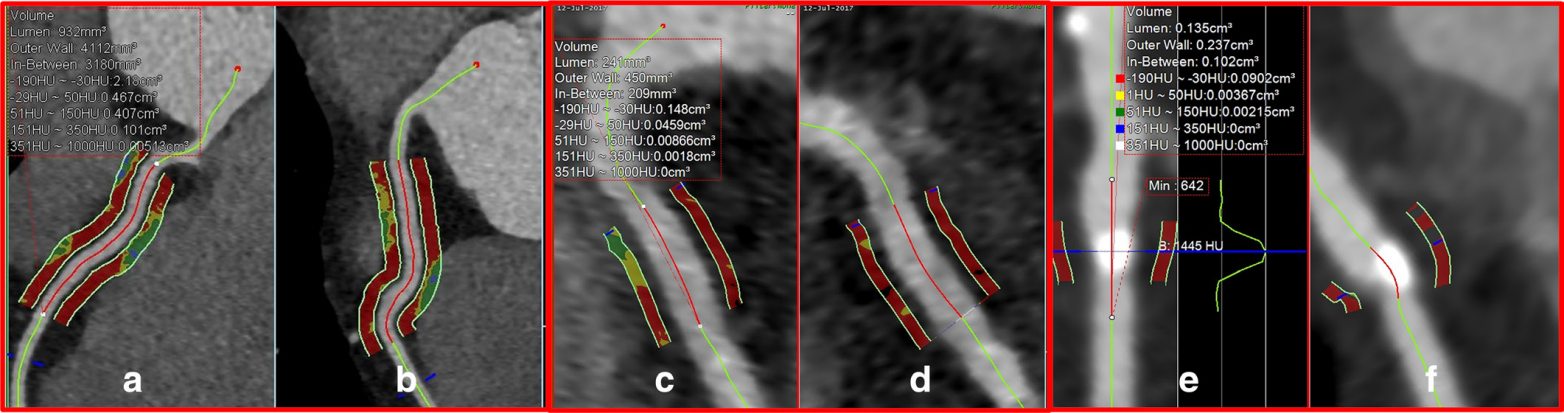
Different measurement approaches for PCAT. (A, B) show the patient-level PCAT measurement. The indicated measurement is made for the RCA with 40 mm length and 3 mm width with 1 mm gap from the coronary lumen. (C, D) show an example of vessel-level PCAT measurement. The measurement is here depicted for RCA with 10 mm length, 1 mm width and 1 mm gap. (E, F) show a lesion-specific PCAT measurement, here across a plaque in the LAD. The measured length is the same as the length of the plaque, while the measured width is 1 mm with 1 mm gap. LAD, left anterior descending; PCAT, pericoronary adipose tissue; RCA, right coronary artery.

Most recent studies have focused on PCAT_MA_ of the RCA as a patient-based biomarker. Patient-based PCAT_MA_ measurement has preference for the evaluation of relationships with patient-level parameters and for patient-based prognostic purposes. In a landmark study by Antonopoulos et al., RCA-based PCAT_MA_ was measured.^26^ The origin point of the measurement was located 10 mm from ostium of the RCA, see Figure 2. A study by Kanaji et al. used the average PCAT_MA_ of all three main coronary arteries with 40 mm measured length and width equivalent to the vessel wall as a patient-based measure instead of the RCA only.^34^ Few studies have investigated the relationship of PCAT_MA_ with patient-based demographic factors such as age or sex. One study by Ma et al in patients without CAD showed that PCAT_MA_ was significantly higher in males than in females.^32^ A study by Yuvaraj et al. also showed that PCAT_MA_ is significantly higher in males than in females independent of the presence of high-risk plaque.^35^ However, this study showed age has no significant effect on PCAT_MA_ while the study by Ma et al. showed that age does have a significant, albeit small, effect.^32^

## Vessel-based measurements

Although patient-based measurements are useful for identifying patient-level disease status and overall risk prediction, many CAD parameters such as stenosis degree and plaque burden are vessel-based. In addition, invasive coronary interventions are vessel-specific.^36,37^ For these associations, vessel-based PCAT_MA_ could be more appropriate and potentially more accurate (Figure 2C and D). Transitioning from patient-based measurements, predominantly using the RCA, to vessel-based measurements using the RCA, left anterior descending artery (LAD) and left circumflex (LCx), requires some methodological changes in order to enable PCAT_MA_ measurement for all three coronaries. The measurement length of 40 mm may cause some issues in proximal LAD and LCX evaluation due to limited length and the presence of side branches. Also, the width requires some adjustments because the presence of veins and myocardium in close vicinity to the coronary arteries may cause artifacts. Finally, because of the different anatomy of coronary arteries, it is important to define and standardize anatomical start and end points of the measurement per coronary.

Ma et al proposed a 10 mm length and 1 mm width, using the original RCA measurement starting point.^32^ This adjusted approach can avoid interference from the myocardium and veins, and is applicable for all three coronaries, see Figure 2C and D. In addition, using comparable lengths and width for each coronary makes measurements more comparable. A study by Balcer et al.^30^ used an even shorter measurement length of 5 mm in order to measure PCAT_MA_ on a per segment level. They showed that PCAT_MA_ was lower in distal segments compared to proximal segments. This indicates that measurement location can influence the PCAT_MA_ value, emphasizing the need for standardized measurement location for vessel-based analysis. For RCA measurements, the most common approach starts 10 mm from the ostium while for LAD and LCX, most studies set the starting point at the bifurcation point of the left coronary artery. One study used 5 mm from the bifurcation as starting point for LAD and LCX measurements.^30^

## Lesion-based measurements

Some studies used lesion-specific methods to investigate specific lesion-related features such as high-risk plaque (HRP) features, plaque composition and hemodynamic significance. Lesion-specific measurement can be of interest specifically when trying to identify plaques at risk of rupture or to identify lesions that will benefit from intervention.^38^ Lesion-based measurements can be performed in different ways, mostly by adjusting measurement lengths, see **Figure 2E & 2F**. The most common approach is to take a PCAT measurement length equivalent to the length of the lesion. However, this causes the lengths of PCAT_MA_ measurements to differ, making comparison between measurements difficult. Another approach is to use a standard measurement length of 10 mm length that covers the lesion. If the lesion is longer than 10 mm, the measurement length could be centered on the mid-point of the lesion. This method uses a uniform measurement length, however in longer lesions information may be incomplete.^39^

## Artificial intelligence and radiomics

With the increasing interest in PCAT and the simultaneous progress in artificial intelligence (AI) and radiomics in the field of radiology, it comes as no surprise that AI and radiomics technology is increasingly used to assess PCAT_MA_.^40^ A detailed overview of the use of AI is given a review by Zhang et al outlining the latest progress of image segmentation, quantification, and application in evaluating cardiac adipose tissue.^40^ In a direct AI approach, mostly convolutional neural networks (CNN) are investigated for segmenting the fat depot and subsequently calculating volume and/or attenuation. No published study so far uses this method for PCAT specifically, but it has been used for EAT.^41^ Another approach uses radiomics to identify PCAT-specific features with a subsequent machine learning approach to create a classifier to identify the outcomes defined.^42,43^ The main study using radiomics and AI for PCAT was performed by Oikonomou et al showing that an AI-powered fat radiomic profile not only identifies inflammatory differences but also improves cardiac risk prediction.^44^ Radiomic analysis could extract more information from PCAT apart from PCAT_MA_. This could potentially improve the risk prediction by PCAT measures while AI-based approaches reduce manual labor resulting by reducing processing time and variability, which could increase the clinical applicability of PCAT analysis.

## PCAT evaluation: imaging technology

### Commonly used tube voltage in CCTA

The currently most recognized PCAT measurement approach is CCTA-derived PCAT_MA_. The proof-of-concept studies of PCAT_MA_ were performed on 120 kVp CCTA scans.^26^ One of the main focuses in CCTA is to lower radiation dose according to the guiding principle of radiation safety, as low as reasonably achievable (ALARA), while maintaining coronary evaluability.^45^ There is a trend of CCTA performed at lower kVp levels. Depending on CT scanner technology, kVp can be lowered to 70 or 80 kVp.^46^ Ma et al, investigating the effect of kVp levels on PCAT_MA_ in patients without CAD, showed that PCAT_MA_ is significantly lower at lower kVp levels (70 kVp:−95.6 HU ± 9.6 *vs*120 kVp:−79.3 HU ± 6.8).^32^ Two other studies confirmed these results, using dual layer spectral CT systems that enable image reconstruction at different keV levels. They showed that PCAT_MA_ measurements at 120 kVp were higher than in 40 keV images in patients with and without CAD. They also suggested that PCAT_MA_ acquired at 40 keV is more closely related to CAD with a higher AUC for identifying significant stenosis compared to 120 keV scans (0.811 *vs* 0.731).^47^ ^48^ With the new photon counting detector CT scanner (PCD-CT), a phantom validation study comparing different energy level from 55 to 80 kev was performed. Results confirm that also with PCD-CT systems, there were significant differences in PCAT_MA_ between different energy levels.^49^ All of these results indicate that for evaluating PCAT_MA_, the kVp level and scan parameters should be considered, especially when quantitative comparisons are made between different scans or with the use of thresholds. Moreover, different spectral characteristics between CT systems may also limit comparisons.

### Non-contrast cardiac CT

Non-contrast cardiac CT is commonly used to quantify coronary calcium. This acquisition is performed without the use of iodine contrast, and at very low radiation dose.^50,51^ These acquisitions are often made earlier at the disease process, and can be used for screening purposes. Adding an early-stage CAD-related biomarker such as PCAT to the traditional calcium score evaluation might improve early identification of patients at risk. Almeida et al, comparing PCAT measurements on contrast-enhanced and non-contrast CT, concluded that PCAT volume showed particularly strong correlation while mean attenuation showed medium correlation.^28^ Balcer et al. studied PCAT volume and attenuation around the proximal and mid segments of the main coronary arteries on non-contrast CT.^30^ They showed that PCAT volume but not attenuation was strongly and independently correlated to culprit lesions.^30^ These studies suggest that non-contrast studies are more suitable for PCAT volume than for attenuation analysis. Further studies of PCAT in non-contrast CT are needed, and better measurement methods of PCAT in non-contrast CT need to be developed.

## PCAT as a CAD biomarker

### PCAT correlation with CAD imaging markers

CTA studies have demonstrated the incremental diagnostic and prognostic value of evaluating plaque features and composition over stenosis severity alone.^52,53^ Recently, several, mostly cross-sectional and small-sized studies showed that PCAT_MA_ is correlated with stenosis severity, plaque burden and HRP presence. See Table 2 for an overview of these studies and the measurement methods they used.

**Table 2.** List of studies reporting the correlation of PCAT_MA_ with imaging markers of CAD severity

First author	Year	Study type	Measurement method	Patients	Main findings
Nakajima^54^	2022	Retrospective cohort study	Patient-based, vessel-based (proximal 40 mm of all coronary arteries) and lesion-based	198 patients presenting with NSTEMI who underwent CCTA prior to intervention	Plaque rupture is associated with higher PCAT_MA_ than plaque erosion both at the culprit plaque level and at the culprit vessel level. The mean PCAT_MA_ of all three coronary arteries is significantly higher in patients with plaque rupture than in plaque erosion.
Yan^55^	2022	Retrospective cohort study	Vessel-based (40 mm segments of the 3 coronary vessels, RCA starting 10 mm distal to the ostium, LAD and LCX starting at LM bifurcation)	247 patients with suspected or known CAD who underwent CCTA (with derived FFRCT) and invasive FFR	PCAT_MA_ predicts ischemia independently of plaque characteristics. The use of a PCAT_MA_ threshold improves discrimination and reclassification abilities of CT visual stenosis assessment compared with stenosis assessment alone. The diagnostic performance of these two methods combined is comparable with FFRCT.
Chen^56^	2021	Retrospective case–control study	Lesion-based	104 patients with chest pain and at least 1 non-calcified plaque on coronary CT divided in two groups: first group with at least one high risk plaque (44 patients), control group with non-high-risk plaques (60 patients)	PCAT_MA_ around high-risk plaques is higher compared to low-risk plaques.
Pasqualetto^57^	2021	Retrospective cohort study	Patient-based (proximal 10–50 mm of RCA and proximal 40 mm of LAD)	202 patients with suspected ACS who underwent pharmacological dipyridamole SE and CCTA within a short interval (<3 months)	PCAT_MA_ is related to coronary microvascular dysfunction detected by pharmacological dipyridamole stress-echocardiography, in particular in patients without obstructive CAD.
Pergola^58^	2021	Retrospective case–control study	Patient-based (proximal 10–50 mm of RCA)	38 patients divided in 3 groups based on CMR findings: myocarditis,^15^ MINOCA^14^ and TTS.^9^ 12 patients with atypical chest pain who underwent CCTA in the control group.	PCAT_MA_ is significantly lower in healthy controls compared to patients with myocarditis, MINOCA and TTS. 8 days after acute event, there is no differences in PCAT_MA_ values between patients with MINOCA and controls.
Yuvaraj^35^	2021	Retrospective case–control study	Patient-based (proximal 10–50 mm ofRCA) and lesion-based	41 CAD patients with HRP matched to 41 CAD patients without HRP	PCAT_MA_ value is higher in stable CAD patients with high-risk plaques compared to those without.
Ma^39^	2021	Retrospective case–control	Vessel-based (proximal 10 mm of LAD, LCX and RCA) Lesion-specific (10 mm in middle of lesion)	165 symptomatic patients with 70 kvp CCTA (93 patients with CAD and 72 patients without CAD)	Lesion-specific PCAT_MA_ is increased in non-calcified and mixed plaque than calcified plaque, and in minimal stenosis compared to severe.
Goeller^59^	2020	Retrospective case–control study	Patient-based (proximal 10–50 mm of RCA)	300 symptomatic patients with suspected CAD from three ethnic groups (100 in each group)	PCAT_MA_ is increased in patients with any plaque in the coronary tree compared to patients without plaque. PCAT_MA_ is correlated with total plaque volume and total plaque burden.
Hoshino^60^	2020	Retrospective cohort study	Patient-based (proximal 40 mm of LAD)	187 patients with intermediate stenosis of the LAD who underwent CCTA and invasive FFR	PCAT_MA_ is associated with CCTA-derived lumen stenosis and plaque size and with functional ischemia as evaluated by FFR.
Lin^43^	2020	Prospective case–control study	Patient-based (proximal 10–50 mm of RCA) and lesion-based	60 prospectively recruited patients with MI who underwent CCTA prior to invasive angiography, matched to patients with stable CAD^61^ and controls with no CAD^61^	PCAT_MA_ independently distinguishes MI from stable CAD and no CAD. Patients with MI have a higher PCAT_MA_ compared with patients with stable CAD and controls.
Yu^38^	2020	Retrospective cohort study	Lesion-based	167 patients with stable angina who underwent CCTA and invasive FFR measurement 2 weeks within	PCAT_MA_ is significantly higher for flow-limiting lesions than for non-flow-limiting lesions. PCAT_MA_ and total plaque volume provide incremental value to diameter stenosis for identifying hemodynamically significant lesions.
Gaibazzi^62^	2019	Retrospective case–control study	Patient-based and vessel-based (40 mm segments of the 3 coronary vessels, RCA starting 10 mm distal to the ostium, LAD and LCX starting at LM bifurcation)	106 patients with MINOCA and Tako-Tsubo Syndrome, who had CCTA and cardiac MRI matched to 106 control subjects with atypical chest pain who had a negative CCTA	In MINOCA and Tako-Tsubo Syndrome, mean PCAT_MA_ demonstrates higher values compared with controls.
Goeller^63^	2019	Retrospective cohort study	Vessel-based (proximal 10–50 mm of RCA)	111 consecutive symptomatic patients with suspected or known CAD and serial CCTA	PCAT_MA_ is related to the progression of plaque burden and helps identifying patients at increased risk of high-risk plaque progression.
Goeller^64^	2018	Retrospective case–control study	Lesion-based	19 patients with ACS matched to 16 controls with stable CAD	PCAT_MA_ is higher around culprit lesions compared with non-culprit lesions of patients with ACS and the lesions of matched controls.

PCAT_MA_: pericoronary adipose tissue mean attenuation; LAD: left anterior descending artery; LCX: left circumflex artery; RCA: right coronary artery; NSTEMI: Non-ST-Elevation Myocardial Infarction; CCTA: Coronary Computed Tomography Angiography; FFR: Fractional Flow Reserve; FFRCT: Fractional Flow Reserve measured with Computed Tomography; CAD: coronary artery disease; MI: Myocardial Infarction; MINOCA: Myocardial Infarction with Non-obstructive Coronary Arteries; ACS: Acute Coronary Syndrome

Ma et al investigated lesion-specific PCAT_MA_ in 165 patients and showed a minimal but significant difference between lesions with a minimal (<25%) and severe stenosis (>70%) (−98.3 HU *vs* −96.2 HU, *p* = 0.037).^39^ Sugiyama et al found in 540 patients that RCA stenoses >50% on ICA have significantly higher PCAT_MA_ compared to patients with a < 50% stenosis (−70.17 HU ± 8.05 *vs* −73.07 HU ± 8.51, *p* < 0.001).^65^ In 2018, Goeller et al. analyzed PCAT_MA_ around every coronary lesion in matched ACS (*n* = 19) and stable CAD (*n* = 16) patients.^64^ They showed that PCAT_MA_was increased around high-burden lesions compared to low-burden ones, within the same patient, both in the ACS group (−69.1 HU *vs* −74.8 HU;*p* = 0.01) and stable CAD group (−69.1 HU *vs* −76.4 HU, *p* = 0.01). Furthermore, the same group found a positive correlation between changes in non-calcified plaque (NCP) burden in the RCA and changes in RCA PCAT_MA_ (*r* = 0.55, *p* < 0.001),^63^ and between RCA-based PCAT_MA_ and total volume and burden of RCA-based NCP (*r* > 0.39, *p* < 0.001).^59^

Multiple studies have demonstrated that CCTA is able to detect plaque features associated with ACS risk.^58,61^ Yuvaraj et al matched 41 patients with stable CAD presenting with HRP on CCTA to 41 patients without HRP. They found that RCA-based PCAT_MA_ was higher in patients with HRP than in patients without (−80.7HU ± 6.50 *vs* −84.2HU ± 8.09 *p* = 0.03).^66^ PCAT_MA_ was also higher in patients with subsequent ACS compared to those without in the whole population (−78.0 HU ± 7.3 *vs* −83.3 HU ± 7.3, *p* = 0.02), and in patients with HRP only (−76.8 HU ± 5.7 *vs* −82.0 HU ± 6.3, *p* = 0.03). Chen et al investigated the correlation between PCAT_MA_and HRP features in 101 patients using spectral CT analyzing 220 plaques of which 48 had HRP features.^56^ They found that PCAT_MA_ around HRP was higher than around non-HRP, especially when using 40 keV images (−119.87 HU ± 22.74 [HRP] *vs* −153.76 HU ± 24.97 [non-HRP], *p* < 0.001). The use of spectral imaging may be interesting considering that in most other studies the differences between different PCAT_MA_ groups are below 10 HU, making this imaging marker hard to use in clinical practice for individual patients.

Regarding the functional significance of coronary artery stenosis, fractional flow reserve (FFR) derived from invasive coronary angiography (ICA) is the gold-standard for guiding revascularization.^67^ Functional measures have shown to be a better predictor of outcome than anatomical assessment of CAD.^68^ Hoshino et al showed in 187 stable patients with intermediate LAD stenosis evaluated by FFR that PCAT_MA_ was associated with CCTA-derived lumen stenosis and FFR-based functional ischemia.^60^ A study by Yan et al involving 247 patients, suggested that vessel-based PCAT_MA_ may predict ischemia identified by FFR, independently of plaque characteristics. The use of a PCAT_MA_ threshold (≥ −71.9 HU) improved discrimination and reclassification abilities of visual stenosis assessment. A combination of PCAT with stenosis severity assessment reached comparable performance as FFRCT (AUC 0.772 *vs* 0.762, *p* = 0.771).^55^ Another study also suggested that PCAT_MA_ may be higher in flow-limiting lesions (FFR≤ 0.8). Lin et al showed that PCAT_MA_ independently distinguishes patients with myocardial infarction (MI) from stable CAD and no CAD (60 subjects for each group). Patients with MI had a higher PCAT_MA_ (−82.3HU  ±  5.5) than patients with stable CAD (−90.6 HU  ±  5.7, *p*  <  0.001) and controls (−95.8 HU  ±  6.2, *p*  <  0.001).^43^

PCAT_MA_ may also have a role in patients without obstructive lesions but with coronary microvascular dysfunction (CMD).^69^ CMD is one of the most recognized causes of myocardial infarction with non-obstructive CAD (≤50% diameter stenosis in major coronary arteries). CMD has been reported in about 50% of patients with chronic coronary syndromes, and up to 20% of those with ACS, in the absence of obstructive coronary lesions.^70^ Pasqualetto and colleagues found a direct relationship between PCAT_MA_ and CMD measured with pharmacological dipyridamolestress-echocardiography, in particular in patients without obstructive CAD.^57^ This suggests that PCAT_MA_ may be related to microvascular dysfunction as well as macrovascular CAD.^57,71^

Gaibazzi et al showed that in MINOCA and Tako-Tsubo Syndrome patients, PCAT_MA_ is increased compared to controls (106 patients in each group), possibly due to differences in coronary artery inflammation.^62^ However, a preliminary report from Pergola et al involving 50 patients showed no differences in PCAT_MA_values between patients with MINOCA and controls, 8 days after an acute event. This may reflect the disappearance of coronary inflammation after MI. Larger studies are needed to identify the potential value of PCAT_MA_ in this subset of patients.

## Risk stratification and prognostication using PCAT

Current cardiac risk stratification relies on traditional clinical risk factors such as age, sex, race, body mass index, hyperlipidemia, hypertension,^72^ and imaging biomarkers such as the coronary calcium score (CCS) measured using CT.^73^ However, coronary calcification represents a non-reversible process that does not regress in response to appropriate medical treatment, limiting its value in secondary prevention.^74^ On the other hand, inflammation has an important role in both atherogenesis and atherosclerotic plaque rupture leading to ACS.^13,75^ Therefore, detection of the inflammatory coronary risk could guide more timely preventive measures in patient care and serve as an early-stage prognosticative marker, see Table 3.

**Table 3.** List of studies reporting the role of PCAT_MA_ in prognostication and risk stratification

First author	Year	Study type	Measurement method	Patients	Main findings	
Ichikawa^76^	2022	Prospective cohort study	Patient based (proximal 10–50 mm of RCA and proximal 40 mm of LAD)	333 patients with Type 2 diabetes mellitus undergoing clinically indicated CCTA	LAD PCATMA > −70.7 HU can significantly predict cardiovascular events in T2DM patients.	
Goeller^77^	2021	Prospective cohort study	Patient based (proximal 10–50 mm of RCA)	293 patients who underwent CCTA because of atypical chest pain and blood sample to analyze serum levels of atherosclerosis-relevant inflammatory mediators	RCA PCAT_MA_ ≥ −73.5 HU is an independent predictor of MACE and shows a weak association with serum levels of atherosclerosis-relevant inflammatory biomarkers.	
Hirano^78^	2021	Prospective cohort study	Patient-based (40 mm segments of the 3 coronary vessels, RCA starting 10 mm distal to the ostium, LAD and LCX starting at LM bifurcation)	114 CAD patients who underwent CCTA and invasive angiography and invasive functional measurements showing intermediate or severe stenosis	PCAT_MA_ is independently and significantly associated with LV mass index in patients with functionally significant epicardial stenosis and preserved systolic function.	
Hoshino^79^	2021	Retrospective cohort study	Patient-based (proximal 40 mm of LAD)	220 consecutive patients with intermediate stenosis who underwent CCTA within 90 days of FFR	Patients with LADPCAT_MA_ ≥ −73.1 HU have an increased risk of MACE.	
Kanaji^34^	2021	Retrospective cohort study	Vessel-based (40 mm segments of the three coronary vessels, RCA starting 10 mm distal to the ostium, LAD and LCX starting at LM bifurcation)	131 patients who underwent CCTA for suspected CAD, showing an intermediate-severe coronary stenosis, followed by phase-contrast MRI prior to PCI within 60 days	PCAT_MA_ is significantly associated with CFR independently of epicardial stenosis severity evaluated by FFR in CAD patients with a single lesion and preserved systolic function.	
Oikonomou^80^	2021	Software training and validation	Patient-based (40 mm segments of the 3 coronary vessels, RCA starting 10 mm distal to the ostium, LAD and LCX starting at LM bifurcation)	3912 consecutive patients undergoing CCTA as part of clinical care in the USA (*n* = 2040, training cohort) and Europe (*n* = 1872, validation cohort).	CT cloud-based quantitative software that calculates PCAT_MA_ and, together with traditional cardiovascular risk factors and information extracted from plaque analysis, gives a vessel-specific coronary inflammation score which correlated with the risk for a fatal cardiac event in the next 8 years.	
Kanaji^36^	2020	Retrospective cohort study	Patient-based (average PCAT_MA_ of LAD, LCX and RCA, 40 mm segments of the 3 coronary vessels, RCA starting 10 mm distal to the ostium, LAD and LCX starting at LM bifurcation)	116 patients with suspected first NSTEMI who underwent CCTA and subsequent successful PCI and CMR	Measured PCAT_MA_ before urgent percutaneous coronary intervention (PCI) was significantly related to lower CFR acquired with magnetic resonance at one-month post‐PCI	
Oikonomou^81^	2018	Retrospective multicohort study	Patient-based (40 mm segments of the 3 coronary vessels, RCA starting 10 mm distal to the ostium, LAD and LCX starting at LM bifurcation). Statistical analysis performed only with RCA PCAT_MA_.	3912 consecutive patients who underwent clinically indicated CCTA in two different institutes. 1872 patients in the first cohort (derivation cohort), 2040 patients in the second cohort (validation cohort).	RCA PCATMA ≥ -70.1 HU predicts all-cause and cardiac mortality beyond current risk stratification approaches, including measurement of coronary calcium and CCTA evaluation.	

PCAT_MA_: pericoronary adipose tissue mean attenuation; LAD: left anterior descending artery; LCX: left circumflex artery; RCA: right coronary artery; CCTA: Coronary Computed Tomography Angiography; FFR: Fractional Flow Reserve; CAD: coronary artery disease; MI: Myocardial Infarction; ACS: Acute Coronary Syndrome; CMR: Cardiac Magnetic Resonance; PCI: Percutaneous Coronary Intervention;CFR: Coronary Flow Reserve; MACE:Major Adverse Cardiovascular Events

Oikonomou et al investigated patient-based PCAT_MA_and its correlation to clinical outcomes in 3912 patients. They showed that high PCAT_MA_ (≥−70.1 HU) could predict all-cause and cardiac mortality better than clinical risk factors and state-of-the-art interpretation of CCTA (HR 9.04, 95% CI: 3.35–24.40, *p* < 0.0001 for cardiac mortality; 2.55, 1.65–3.92, *p* < 0.0001 for all-cause mortality).^81^ In a study by Hoshino et al including 220 consecutive patients with intermediate stenosis, the authors showed that a PCAT_MA_≥−73.1 HU was related to an increased risk of major adverse cardiac events (MACE) (multivariate HR 3.11, 95% CI:1.40–6.94, *p* = 0.005). Goeller et al defined a similar cut-off value of −73.5 HU for PCAT_MA_ as independent predictor of MACE (HR 2.01, *p* = 0.044).^77^

Oikonomou et al used a cloud-based quantitative software that calculates vessel-based PCAT_MA_, and incorporates those values into a risk prediction algorithm together with traditional cardiovascular risk factors and information extracted from CCTA-based plaque analysis. The result was a vessel-specific coronary inflammation score that correlated with the 8 year risk for a fatal cardiac event (∆C statistic of 0.085, *p* = 0.01 for the US Cohort and ∆C of 0.149, *p* < 0.001 in the European cohort).^80^

Some studies focused on the relationship between PCAT_MA_ and outcome in specific high-risk subgroups. Ichikawa et al analyzed 333 patients with Type 2 diabetes, finding that high LAD-derived PCAT_MA_ predicted MACE.^76^ Left ventricular hypertrophy (LVH) is a powerful predictor for cardiac disease prognosis. Hirano et al investigated 114 CAD patients with intermediate or severe stenosis on CCTA and invasive physiological tests. They found an independent relationship between PCAT_MA_ and LV mass index in patients with functionally significant CAD and preserved systolic function.^78^

Coronary flow reserve (CFR) is the ratio of myocardial blood flow at stress to myocardial blood flow at rest. The presence of significant stenosis changes this parameter, making it an outcome predictor in patients with CAD. Kanaji et al. performed a retrospective cohort study involving 116 patients with non-ST segment elevation MI and successful coronary intervention and cardiac MRI in addition to CCTA imaging. They showed that PCAT_MA_ measured before urgent PCI was related to lower CFR based on cardiac MRI at 1-month post-PCI.^36^ The same author demonstrated a relationship between PCAT_MA_ and CFR independent of FFR in CAD patients with a single lesion and preserved systolic function.^34^

## Conclusion

CT-based PCAT analysis enables non-invasive evaluation of coronary inflammation, which is an indicator of cardiac disease, with PCAT_MA_ emerging as the most valuable measurement. Multiple studies have found a relationship between PCAT_MA_ and presence and severity of CAD and risk of cardiac events. Integrating PCAT_MA_ into modern coronary CTA interpretation may in the future help in the identification of individuals at risk of MACE, who might be candidates for more intensive treatment. This review shows that there are still limitations and gaps of knowledge in the literature regarding PCAT evaluation. One of the main limitations this review points out is the variation in measurement approaches. In order to move forward, the measurement methodology should be standardized, considering influences such as age, sex, lumen-normalization, and tube voltage. Although PCAT_MA_ is currently mostly acquired in CCTA images, non-contrast cardiac CT-derived PCAT_MA_ may be a potential direction for evaluation at an early disease stage. Moreover, whether PCAT volume is relevant needs further exploration. Multicenter and prospective studies are needed to find cut-off values of PCAT_MA_ for diagnostic and prognostic purposes. Finally, so far, PCAT_MA_ merely shows associations on group level with limited HU differences (around 3–10 HU). Whether PCAT_MA_, in view of physiological variations, measurement variations, and small absolute differences, can have value for individual patients, will need to be determined.

## References

[1] Hansson GK . Inflammation, atherosclerosis, and coronary artery disease. N Engl J Med 2005; 352: 1685–95. doi: 10.1056/NEJMra043430 15843671

[2] Antoniades C, Antonopoulos AS, Deanfield J . Imaging residual inflammatory cardiovascular risk. Eur Heart J 2020; 41: 748–58. doi: 10.1093/eurheartj/ehz474 31317172

[3] Goeller M, Achenbach S, Duncker H, Dey D, Marwan M . Imaging of the pericoronary adipose tissue (PCAT) using cardiac computed tomography: modern clinical implications. J Thorac Imaging 2021; 36: 149–61. doi: 10.1097/RTI.0000000000000583 33875629

[4] Shuster A, Patlas M, Pinthus JH, Mourtzakis M . The clinical importance of visceral adiposity: a critical review of methods for visceral adipose tissue analysis. Br J Radiol 2012; 85: 1–10. doi: 10.1259/bjr/38447238 21937614 PMC3473928

[5] Ansaldo AM, Montecucco F, Sahebkar A, Dallegri F, Carbone F . Epicardial adipose tissue and cardiovascular diseases. Int J Cardiol 2019; 278: 254–60. doi: 10.1016/j.ijcard.2018.09.089 30297191

[6] Cherian S, Lopaschuk GD, Carvalho E . Cellular cross-talk between epicardial adipose tissue and myocardium in relation to the pathogenesis of cardiovascular disease. Am J Physiol Endocrinol Metab 2012; 303: E937–49. doi: 10.1152/ajpendo.00061.2012 22895783

[7] Packer M . Epicardial adipose tissue may mediate deleterious effects of obesity and inflammation on the myocardium. J Am Coll Cardiol 2018; 71: 2360–72. doi: 10.1016/j.jacc.2018.03.509 29773163

[8] Kim SA, Kim MN, Shim WJ, Park SM . Epicardial adipose tissue is related to cardiac function in elderly women, but not in men. Nutr Metab Cardiovasc Dis 2017; 27: 41–47. doi: 10.1016/j.numecd.2016.11.001 27988072

[9] Iacobellis G, Corradi D, Sharma AM . Epicardial adipose tissue: anatomic, biomolecular and clinical relationships with the heart. Nat Clin Pract Cardiovasc Med 2005; 2: 536–43. doi: 10.1038/ncpcardio0319 16186852

[10] Rabkin SW . Epicardial fat: properties, function and relationship to obesity. Obes Rev 2007; 8: 253–61. doi: 10.1111/j.1467-789X.2006.00293.x 17444966

[11] Furuhashi M, Fuseya T, Murata M, Hoshina K, Ishimura S, Mita T, et al . Local production of fatty acid-binding protein 4 in epicardial/perivascular fat and macrophages is linked to coronary atherosclerosis. Arterioscler Thromb Vasc Biol 2016; 36: 825–34. doi: 10.1161/ATVBAHA.116.307225 27013610

[12] Meijer RI, Serné EH, Korkmaz HI, van der Peet DL, de Boer MP, Niessen HWM, et al . Insulin-Induced changes in skeletal muscle microvascular perfusion are dependent upon perivascular adipose tissue in women. Diabetologia 2015; 58: 1907–15. doi: 10.1007/s00125-015-3606-8 26003324 PMC4499111

[13] Libby P, Ridker PM, Hansson GK, Leducq Transatlantic Network on Atherothrombosis . Inflammation in atherosclerosis: from pathophysiology to practice. J Am Coll Cardiol 2009; 54: 2129–38. doi: 10.1016/j.jacc.2009.09.009 19942084 PMC2834169

[14] Berg G, Miksztowicz V, Morales C, Barchuk M . Epicardial adipose tissue in cardiovascular disease. In: In: Advances in Experimental Medicine and Biology. New York LLC: Springer; 2019, pp. 131–43. 10.1007/978-3-030-11488-6_9 31140176

[15] Park J-S, Choi S-Y, Zheng M, Yang H-M, Lim H-S, Choi B-J, et al . Epicardial adipose tissue thickness is a predictor for plaque vulnerability in patients with significant coronary artery disease. Atherosclerosis 2013; 226: 134–39. doi: 10.1016/j.atherosclerosis.2012.11.001 23206980

[16] Mancio J, Azevedo D, Saraiva F, Azevedo AI, Pires-Morais G, Leite-Moreira A, et al . Epicardial adipose tissue volume assessed by computed tomography and coronary artery disease: a systematic review and meta-analysis. Eur Heart J Cardiovasc Imaging 2018; 19: 490–97. doi: 10.1093/ehjci/jex314 29236951

[17] Nerlekar N, Baey YW, Brown AJ, Muthalaly RG, Dey D, Tamarappoo B, et al . n.d.).( Poor correlation, reproducibility, and agreement between volumetric versus linear epicardial adipose tissue measurement: A 3D computed tomography versus 2D echocardiography comparison. JACC: Cardiovascular Imaging 2018; 11. 10.1016/j.jcmg.2017.10.019 PMC6035093 29361482

[18] Rostamzadeh A, Khademvatani K, Seyed Mohammadzadeh MH, Ashori S, Hajahmadi Poorrafsanjani M, Rahimi B, et al . Association of epicardial fat thickness assessed by echocardiography with the severity of coronary artery disease. J Cardiovasc Thorac Res 2020; 12: 114–19. doi: 10.34172/jcvtr.2020.19 32626551 PMC7321005

[19] Vach M, Luetkens JA, Faron A, Isaak A, Salam B, Thomas D, et al . Association between single-slice and whole heart measurements of epicardial and pericardial fat in cardiac MRI. Acta Radiol 2021; 2841851211054192. doi: 10.1177/02841851211054192 34747661

[20] Archer JM, Raggi P, Amin SB, Zhang C, Gadiyaram V, Stillman AE . Season and clinical factors influence epicardial adipose tissue attenuation measurement on computed tomography and may hamper its utilization as a risk marker. Atherosclerosis 2021; 321: 8–13. doi: 10.1016/j.atherosclerosis.2021.01.025 33588217

[21] Honold S, Wildauer M, Beyer C, Feuchtner G, Senoner T, Jaschke W, et al . Reciprocal communication of pericoronary adipose tissue and coronary atherogenesis. Eur J Radiol 2021; 136: 109531. doi: 10.1016/j.ejrad.2021.109531 33486436

[22] Akoumianakis I, Antoniades C . The interplay between adipose tissue and the cardiovascular system: is fat always bad? Cardiovasc Res 2017; 113: 999–1008. doi: 10.1093/cvr/cvx111 28582523

[23] Antonopoulos AS, Sanna F, Sabharwal N, Thomas S, Oikonomou EK, Herdman L, et al . Detecting human coronary inflammation by imaging perivascular fat. Sci Transl Med 2017; 9(398): eaal2658. doi: 10.1126/scitranslmed.aal2658 28701474

[24] Margaritis M, Antonopoulos AS, Digby J, Lee R, Reilly S, Coutinho P, et al . Interactions between vascular wall and perivascular adipose tissue reveal novel roles for adiponectin in the regulation of endothelial nitric oxide synthase function in human vessels. Circulation 2013; 127: 2209–21: 22. doi: 10.1161/CIRCULATIONAHA.112.001133 23625959

[25] Margaritis M, Antonopoulos AS, Digby J, Lee R, Reilly S, Coutinho P, et al . Interactions between vascular wall and perivascular adipose tissue reveal novel roles for adiponectin in the regulation of endothelial nitric oxide synthase function in human vessels. Circulation 2013; 127: 2209–21. doi: 10.1161/CIRCULATIONAHA.112.001133 23625959

[26] Antonopoulos AS, Sanna F, Sabharwal N, Thomas S, Oikonomou EK, Herdman L, et al . Detecting human coronary inflammation by imaging perivascular fat. Sci Transl Med 2017; 9: 398: eaal2658. doi: 10.1126/scitranslmed.aal2658 28701474

[27] Qi XY, Qu SL, Xiong WH, Rom O, Chang L, Jiang ZS . n.d.).( Perivascular adipose tissue (PVAT) in atherosclerosis: A double-edged sword. Cardiovascular Diabetology 2018; 17. 10.1186/s12933-018-0777-x PMC6180425 30305178

[28] Almeida S, Pelter M, Shaikh K, Cherukuri L, Birudaraju D, Kim K, et al . Feasibility of measuring pericoronary fat from precontrast scans: effect of iodinated contrast on pericoronary fat attenuation. J Cardiovasc Comput Tomogr 2020; 14: 490–94. doi: 10.1016/j.jcct.2020.04.004 32576456

[29] Relationship of thoracic fat depots with coronary atherosclerosis and circulating inflammatory biomarkers. Obesity 2015; 23(6). 10.1002/oby.21080 PMC4446160 25960369

[30] Balcer B, Dykun I, Schlosser T, Forsting M, Rassaf T, Mahabadi AA . Pericoronary fat volume but not attenuation differentiates culprit lesions in patients with myocardial infarction. Atherosclerosis 2018; 276: 182–88. doi: 10.1016/j.atherosclerosis.2018.05.035 29866393

[31] Sun JT, Sheng XC, Feng Q, Yin Y, Li Z, Ding S, et al . Pericoronary fat attenuation index is associated with vulnerable plaque components and local immune-inflammatory activation in patients with non-ST elevation acute coronary syndrome. J Am Heart Assoc 2022; 11(2): e022879. doi: 10.1161/JAHA.121.022879 35023363 PMC9238519

[32] Ma R, Ties D, van Assen M, Pelgrim GJ, Sidorenkov G, van Ooijen PMA, et al . Towards reference values of pericoronary adipose tissue attenuation: impact of coronary artery and tube voltage in coronary computed tomography angiography. Eur Radiol 2020; 30: 6838–46. doi: 10.1007/s00330-020-07069-0 32700017 PMC7599179

[33] Chatterjee D, Shou BL, Matheson MB, Ostovaneh MR, Rochitte C, Chen MY, et al . Perivascular fat attenuation for predicting adverse cardiac events in stable patients undergoing invasive coronary angiography. J Cardiovasc Comput Tomogr 2022; 16: 483–90. doi: 10.1016/j.jcct.2022.05.004 35680534 PMC9684349

[34] Kanaji Y, Sugiyama T, Hoshino M, Misawa T, Nagamine T, Yasui Y, et al . Physiological significance of pericoronary inflammation in epicardial functional stenosis and global coronary flow reserve. Sci Rep 2021; 11(1): 19026. doi: 10.1038/s41598-021-97849-5 34561466 PMC8463533

[35] Yuvaraj J, Lin A, Nerlekar N, Munnur RK, Cameron JD, Dey D, et al . Pericoronary adipose tissue attenuation is associated with high-risk plaque and subsequent acute coronary syndrome in patients with stable coronary artery disease. Cells 2021; 10(5): 1143. doi: 10.3390/cells10051143 34068518 PMC8150579

[36] Kanaji Y, Hirano H, Sugiyama T, Hoshino M, Horie T, Misawa T, et al . Pre‐percutaneous coronary intervention pericoronary adipose tissue attenuation evaluated by computed tomography predicts global coronary flow reserve after urgent revascularization in patients with non–st‐segment–elevation acute coronary syndrome. JAHA 2020; 9: 17. doi: 10.1161/JAHA.120.016504 PMC7660767 32856503

[37] Bengs S, Haider A, Warnock GI, Fiechter M, Pargaetzi Y, Rampidis G, et al . Quantification of perivascular inflammation does not provide incremental prognostic value over myocardial perfusion imaging and calcium scoring. Eur J Nucl Med Mol Imaging 2021; 48: 1806–12. doi: 10.1007/s00259-020-05106-0 33200300 PMC8113311

[38] Yu M, Dai X, Deng J, Lu Z, Shen C, Zhang J . Diagnostic performance of perivascular fat attenuation index to predict hemodynamic significance of coronary stenosis: a preliminary coronary computed tomography angiography study. Eur Radiol 2020; 30: 673–81. doi: 10.1007/s00330-019-06400-8 31444596

[39] Ma R, van Assen M, Ties D, Pelgrim GJ, van Dijk R, Sidorenkov G, et al . Focal pericoronary adipose tissue attenuation is related to plaque presence, plaque type, and stenosis severity in coronary cta. Eur Radiol 2021; 31: 7251–61. doi: 10.1007/s00330-021-07882-1 33860371 PMC8452552

[40] Zhang L, Sun J, Jiang B, Wang L, Zhang Y, Xie X . Development of artificial intelligence in epicardial and pericoronary adipose tissue imaging: a systematic review. Eur J Hybrid Imaging 2021; 5(1): 14. doi: 10.1186/s41824-021-00107-0 34312735 PMC8313612

[41] Liu L, Ma R, Ooijen P van, Oudkerk M, Vliegenthart R, Brune C, et al . n.d.).( Using the U-Net Family for Epicardial Adipose Tissue Segmentation and Quantification in Non-Contrast CT. In Review. doi: 10.21203/rs.3.rs-1530148/v1

[42] Wen D, Xu Z, An R, Ren J, Jia Y, Li J, et al . Predicting haemodynamic significance of coronary stenosis with radiomics-based pericoronary adipose tissue characteristics. Clin Radiol 2022; 77: e154–61. doi: 10.1016/j.crad.2021.10.019 34852918

[43] Lin A, Kolossváry M, Yuvaraj J, Cadet S, McElhinney PA, Jiang C, et al. Myocardial Infarction Associates With a Distinct Pericoronary Adipose Tissue Radiomic Phenotype: A Prospective Case-Control Study. JACC Cardiovasc Imaging. 2020;13(11). 10.1016/j.jcmg.2020.06.033 PMC7996075 32861654

[44] Oikonomou EK, Williams MC, Kotanidis CP, Desai MY, Marwan M, Antonopoulos AS, et al . A novel machine learning-derived radiotranscriptomic signature of perivascular fat improves cardiac risk prediction using coronary CT angiography. Eur Heart J 2019; 40: 43. 10.1093/eurheartj/ehz592 PMC6855141 31504423

[45] Gimelli A, Achenbach S, Buechel RR, Edvardsen T, Francone M, Gaemperli O, et al . Strategies for radiation dose reduction in nuclear cardiology and cardiac computed tomography imaging: A report from the european association of cardiovascular imaging (EACVI). The Cardiovascular Committee of European Association of Nuclear Medicine (EANM), and the European Society of Cardiovascular Radiology (ESCR) European Heart Journal 2018; 39. 10.1093/eurheartj/ehx582 29059384

[46] Hell MM, Bittner D, Schuhbaeck A, Muschiol G, Brand M, Lell M, et al . Prospectively ECG-triggered high-pitch coronary angiography with third-generation dual-source CT at 70 kvp tube voltage: feasibility, image quality, radiation dose, and effect of iterative reconstruction. Journal of Cardiovascular Computed Tomography 2014; 8: 418–25. doi: 10.1016/j.jcct.2014.09.003 25439789

[47] Dang Y, Chen X, Ma S, Ma Y, Ma Q, Zhou K, et al . Association of pericoronary adipose tissue quality determined by dual-layer spectral detector CT with severity of coronary artery disease: a preliminary study. Front Cardiovasc Med 2021; 8: 720127. doi: 10.3389/fcvm.2021.720127 34660721 PMC8514719

[48] Zhu X, Chen X, Ma S, Zhou K, Hou Y . Dual-layer spectral detector CT to study the correlation between pericoronary adipose tissue and coronary artery stenosis. J Cardiothorac Surg 2021; 16(1): 325. doi: 10.1186/s13019-021-01709-2 34743735 PMC8574033

[49] Mergen V, Ried E, Allmendinger T, Sartoretti T, Higashigaito K, Manka R, et al . Epicardial adipose tissue attenuation and fat attenuation index: phantom study and in vivo measurements with photon-counting detector CT. AJR Am J Roentgenol 2022; 218: 822–29. doi: 10.2214/AJR.21.26930 34877869

[50] Xu L, Xu Y, Coulden R, Sonnex E, Hrybouski S, Paterson I, et al . Comparison of epicardial adipose tissue radiodensity threshold between contrast and non-contrast enhanced computed tomography scans: a cohort study of derivation and validation. Atherosclerosis 2018; 275: 74–79. doi: 10.1016/j.atherosclerosis.2018.05.013 29864608

[51] Marwan M, Koenig S, Schreiber K, Ammon F, Goeller M, Bittner D, et al . Quantification of epicardial adipose tissue by cardiac CT: influence of acquisition parameters and contrast enhancement. Eur J Radiol 2019; 121: 108732. doi: 10.1016/j.ejrad.2019.108732 31711022

[52] Kolossváry M, Szilveszter B, Merkely B, Maurovich-Horvat P . Plaque imaging with CT-a comprehensive review on coronary CT angiography based risk assessment. Cardiovasc Diagn Ther 2017; 7: 489–506. doi: 10.21037/cdt.2016.11.06 29255692 PMC5716945

[53] Williams MC, Moss AJ, Dweck M, Adamson PD, Alam S, Hunter A, et al . Coronary artery plaque characteristics associated with adverse outcomes in the SCOT-HEART study. J Am Coll Cardiol 2019; 73: 291–301. doi: 10.1016/j.jacc.2018.10.066 30678759 PMC6342893

[54] Nakajima A, Sugiyama T, Araki M, Seegers LM, Dey D, McNulty I, et al . Plaque rupture, compared with plaque erosion, is associated with a higher level of pancoronary inflammation. JACC Cardiovasc Imaging 2022; 15: 828–39. doi: 10.1016/j.jcmg.2021.10.014 34876381 PMC12918542

[55] Yan H, Zhao N, Geng W, Hou Z, Gao Y, Lu B . Pericoronary fat attenuation index and coronary plaque quantified from coronary computed tomography angiography identify ischemia-causing lesions. International Journal of Cardiology 2022; 357: 8–13. doi: 10.1016/j.ijcard.2022.03.033 35306030

[56] Chen X, Dang Y, Hu H, Ma S, Ma Y, Wang K, et al . Pericoronary adipose tissue attenuation assessed by dual-layer spectral detector computed tomography is a sensitive imaging marker of high-risk plaques. Quant Imaging Med Surg 2021; 11: 2093–2103. doi: 10.21037/qims-20-860 33936990 PMC8047372

[57] Pasqualetto MC, Tuttolomondo D, Cutruzzolà A, Niccoli G, Dey D, Greco A, et al . Human coronary inflammation by computed tomography: relationship with coronary microvascular dysfunction. International Journal of Cardiology 2021; 336: 8–13. doi: 10.1016/j.ijcard.2021.05.040 34052238

[58] Pergola V, Previtero M, Cecere A, Storer V, Castiello T, Baritussio A, et al . Clinical value and time course of pericoronary fat inflammation in patients with angiographically nonobstructive coronaries: a preliminary report. J Clin Med 2021; 10(8): 1786. doi: 10.3390/jcm10081786 33924006 PMC8073076

[59] Goeller M, Rahman Ihdayhid A, Cadet S, Lin A, Adams D, Thakur U, et al . Pericoronary adipose tissue and quantitative global non-calcified plaque characteristics from CT angiography do not differ in matched south asian, east asian and european-origin caucasian patients with stable chest pain. Eur J Radiol 2020; 125: 108874. doi: 10.1016/j.ejrad.2020.108874 32087467 PMC8444621

[60] Hoshino M, Yang S, Sugiyama T, Zhang J, Kanaji Y, Yamaguchi M, et al . Peri-coronary inflammation is associated with findings on coronary computed tomography angiography and fractional flow reserve. J CardiovascComputTomogr. 2020;14(6). 10.1016/j.jcct.2020.02.002 32057707

[61] Dawson LP, Layland J . High-Risk coronary plaque features: a narrative review. Cardiol Ther 2022; 11: 319–35. doi: 10.1007/s40119-022-00271-9 35731471 PMC9381667

[62] Gaibazzi N, Martini C, Botti A, Pinazzi A, Bottazzi B, Palumbo AA . Coronary inflammation by computed tomography pericoronary fat attenuation in MINOCA and tako-tsubo syndrome. J Am Heart Assoc 2019; 8: e013235. doi: 10.1161/JAHA.119.013235 31462127 PMC6755824

[63] Goeller M, Tamarappoo BK, Kwan AC, Cadet S, Commandeur F, Razipour A, et al . Relationship between changes in pericoronary adipose tissue attenuation and coronary plaque burden quantified from coronary computed tomography angiography. Eur Heart J Cardiovasc Imaging 2019; 20: 636–43. doi: 10.1093/ehjci/jez013 30789223 PMC6821274

[64] Goeller M, Achenbach S, Cadet S, Kwan AC, Commandeur F, Slomka PJ, et al . Pericoronary adipose tissue computed tomography attenuation and high-risk plaque characteristics in acute coronary syndrome compared with stable coronary artery disease. JAMA Cardiol 2018; 3: 858–63. doi: 10.1001/jamacardio.2018.1997 30027285 PMC6233643

[65] Sugiyama T, Kanaji Y, Hoshino M, Yamaguchi M, Hada M, Ohya H, et al . Determinants of pericoronary adipose tissue attenuation on computed tomography angiography in coronary artery disease. J Am Heart Assoc 2020; 9: e016202. doi: 10.1161/JAHA.120.016202 32750306 PMC7792233

[66] Yuvaraj J, Lin A, Nerlekar N, Munnur RK, Cameron JD, Dey D, et al . Pericoronary adipose tissue attenuation is associated with high-risk plaque and subsequent acute coronary syndrome in patients with stable coronary artery disease. Cells 2021; 10: 1143. doi: 10.3390/cells10051143 34068518 PMC8150579

[67] De Bruyne B, Fearon WF, Pijls NHJ, Barbato E, Tonino P, Piroth Z, et al . Fractional flow reserve-guided PCI for stable coronary artery disease. N Engl J Med 2014; 371: 1208–17. doi: 10.1056/NEJMoa1408758 25176289

[68] Wardziak Ł, Kruk M, Pleban W, Demkow M, Rużyłło W, Dzielińska Z, et al . Coronary cta enhanced with cta based FFR analysis provides higher diagnostic value than invasive coronary angiography in patients with intermediate coronary stenosis. Journal of Cardiovascular Computed Tomography 2019; 13: 62–67. doi: 10.1016/j.jcct.2018.10.004 30309764

[69] Vancheri F, Longo G, Vancheri S, Henein M . Coronary Microvascular Dysfunction J Clin Med 2020; 6;9(9):2880. 10.3390/jcm9092880 PMC7563453 32899944

[70] Hoshino M, Yang S, Sugiyama T, Zhang J, Kanaji Y, Hamaya R, et al . Characteristic findings of microvascular dysfunction on coronary computed tomography angiography in patients with intermediate coronary stenosis. Eur Radiol 2021; 31: 9198–9210. doi: 10.1007/s00330-021-07909-7 34009414

[71] Hoshino M, Yang S, Sugiyama T, Zhang J, Kanaji Y, Hamaya R, et al . Characteristic findings of microvascular dysfunction on coronary computed tomography angiography in patients with intermediate coronary stenosis. Eur Radiol 2021; 31: 9198–9210. doi: 10.1007/s00330-021-07909-7 34009414

[72] Hajar R . Risk factors for coronary artery disease: historical perspectives. Heart Views 2017; 18: 109–14. doi: 10.4103/HEARTVIEWS.HEARTVIEWS_106_17 29184622 PMC5686931

[73] Greenland P, LaBree L, Azen SP, Doherty TM, Detrano RC . Coronary artery calcium score combined with Framingham score for risk prediction in asymptomatic individuals. JAMA 2004; 291: 210–15. doi: 10.1001/jama.291.2.210 14722147

[74] Alexopoulos N, Melek BH, Arepalli CD, Hartlage G-R, Chen Z, Kim S, et al . Effect of intensive versus moderate lipid-lowering therapy on epicardial adipose tissue in hyperlipidemic post-menopausal women. Journal of the American College of Cardiology 2013; 61: 1956–61. doi: 10.1016/j.jacc.2012.12.051 23500254

[75] Wolf D, Ley K . Immunity and inflammation in atherosclerosis. Circ Res 2019; 124: 315–27. doi: 10.1161/CIRCRESAHA.118.313591 30653442 PMC6342482

[76] Ichikawa K, Miyoshi T, Osawa K, Nakashima M, Miki T, Nishihara T, et al . High pericoronary adipose tissue attenuation on computed tomography angiography predicts cardiovascular events in patients with type 2 diabetes mellitus: post-hoc analysis from a prospective cohort study. Cardiovasc Diabetol 2022; 21(1. doi: 10.1186/s12933-022-01478-9 PMC8933955 35303857

[77] Goeller M, Achenbach S, Herrmann N, Bittner DO, Kilian T, Dey D, et al . Pericoronary adipose tissue CT attenuation and its association with serum levels of atherosclerosis-relevant inflammatory mediators, coronary calcification and major adverse cardiac events. Journal of Cardiovascular Computed Tomography 2021; 15: 449–54. doi: 10.1016/j.jcct.2021.03.005 33867303

[78] Hirano H, Kanaji Y, Sugiyama T, Hoshino M, Horie T, Misawa T, et al . Impact of pericoronary adipose tissue inflammation on left ventricular hypertrophy and regional physiological indices in stable coronary artery disease patients with preserved systolic function. Heart Vessels 2021; 36: 24–37. doi: 10.1007/s00380-020-01658-1 32638076

[79] Hoshino M, Zhang J, Sugiyama T, Yang S, Kanaji Y, Hamaya R, et al . Prognostic value of pericoronary inflammation and unsupervised machine-learning-defined phenotypic clustering of CT angiographic findings. Int J Cardiol 2021; 333: 226–32. doi: 10.1016/j.ijcard.2021.03.019 33741428

[80] Oikonomou EK, Antonopoulos AS, Schottlander D, Marwan M, Mathers C, Tomlins P, et al . Standardized measurement of coronary inflammation using cardiovascular computed tomography: integration in clinical care as a prognostic medical device. Cardiovasc Res 2021; 117: 13. doi: 10.1093/cvr/cvab286 34450625

[81] Oikonomou EK, Marwan M, Desai MY, Mancio J, Alashi A, Hutt Centeno E, et al . Non-Invasive detection of coronary inflammation using computed tomography and prediction of residual cardiovascular risk (the CRISP CT study): a post-hoc analysis of prospective outcome data. The Lancet 2018; 392: 929–39. doi: 10.1016/S0140-6736(18)31114-0 PMC6137540 30170852

